# Anti-biofilm and Antibacterial Activities of Silver Nanoparticles Synthesized by the Reducing Activity of Phytoconstituents Present in the Indian Medicinal Plants

**DOI:** 10.3389/fmicb.2020.01143

**Published:** 2020-06-23

**Authors:** Yugal Kishore Mohanta, Kunal Biswas, Santosh Kumar Jena, Abeer Hashem, Elsayed Fathi Abd_Allah, Tapan Kumar Mohanta

**Affiliations:** ^1^Department of Botany, North Orissa University, Baripada, India; ^2^Department of Biotechnology, Maulana Abul Kalam Azad University of Technology, Haringhata, India; ^3^Department of Biotechnology, North Orissa University, Baripada, India; ^4^Botany and Microbiology Department, College of Science, King Saud University, Riyadh, Saudi Arabia; ^5^Mycology and Plant Disease Survey Department, Plant Pathology Research Institute, Agriculture Research Center, Giza, Egypt; ^6^Plant Production Department, College of Food & Agricultural Sciences, King Saud University, Riyadh, Saudi Arabia; ^7^Natural and Medical Sciences Research Center, University of Nizwa, Nizwa, Oman

**Keywords:** phyto-synthesis, silver nanoparticles, medicinal plants, anti-bacterial activity, anti-biofilm activity

## Abstract

Biofilm forming from a variety of microbial pathogens can pose a serious health hazard that is difficult to combat. Nanotechnology, however, represents a new approach to fighting and eradicating biofilm-forming microorganisms. In the present study, the sustainable synthesis and characterization of biocompatible silver nanoparticles (AgNPs) from leaf extracts of *Semecarpus anacardium*, *Glochidion lanceolarium*, and *Bridelia retusa* was explored. Continuous synthesis was observed in a UV–vis spectroscopic analysis and the participating phytoconstituents, flavonoids, phenolic compounds, phytosterols, and glycosides, were characterized by Attenuated total reflectance-Fourier transform infrared spectroscopy. The size and surface charge of the particles were also measured by dynamic light scattering spectroscopy. Scanning electron microscopy study was employed to examine the morphology of the nanoparticles. The spectroscopic and microscopic study confirmed the successful synthesis of AgNPs by plant extracts acting as strong reducing agents. The synthesized AgNPs were screened for antibacterial and anti-biofilm activity against human pathogens *Pseudomonas aeruginosa, Escherichia coli*, and *Staphylococcus aureus*. Results of the study demonstrate the potential of phyto-synthesized AgNPs to act as anti-biofilm agents and for other biomedical applications.

## Introduction

Nanotechnology is a multidisciplinary science focused on the wide-ranging properties of nanoparticles. Nanoparticles exhibit a broad range of physicochemical properties that greatly contrast their bulk analogs. Nanoparticles in the size range of 1-100 nm display unique and novel properties (Jeevanandam et al., 2018; Zia-ur-Rehman et al., 2018; Teulon et al., 2019). Nanoscale materials exhibit their unique properties due to their high surface energy, large proportion of surface atoms, low level of imperfection, and spatial confinement (Bai et al., 2009). Nanoparticles have distinct advantages over bulk materials due to their surface plasmon light scattering, surface plasmon resonance (SPR), surface-enhanced Rayleigh scattering, and surface-enhanced Raman scattering (SERS) properties (Jain et al., 2007). Due to their unique features, nanoscale materials can be used as building blocks for various optoelectronics, electronics, chemical sensing, and biological applications (Ramanavicius et al., 2005).

Silver is recognized for its anti-microbial activity against a broad spectrum of pathogenic microorganisms (Mohanta et al., 2016b). Silver (Ag) has been used since ancient times for its medicinal properties and now the activity and application of silver nanoparticles (AgNPs) is being explored in medical research. Topical antimicrobial ointments and creams contain silver to prevent microbial infection of burns and open wounds. Silver is also commonly used in medical devices and implants that are manufactured with silver-impregnated polymers. In addition, several silver-containing consumer products, such as colloidal silver gel and silver-embedded fabrics, are now used in sporting equipment (Kim et al., 2007; Song and Kim, 2009).

The easiest and most convenient approach for the rapid synthesis of AgNPs, which has been used for decades, is by a chemical synthesis process. The chemical approach to AgNP synthesis, however, involves the use of several toxic reagents. As several metal nanoparticles, such as gold, platinum, and silver, are frequently used in ointments, creams, cosmetics, etc., that interface with human skin, methods of nanoparticle synthesis that have low toxicity and are eco-friendly need to be explored and developed (Nayak et al., 2015). Several biological approaches for the synthesis of metal nanoparticles have been proposed, including the use of microorganisms (Narayanan and Sakthivel, 2010) and plants (Mittal et al., 2013). Plant-mediated synthesis of nanoparticles is highly attractive as it requires less effort than maintaining and culturing microorganisms. Plants’ biomolecules are natural and typically exhibit low toxicity to living cells which makes the use of plant metabolites in the synthesis of AgNPs favorable (Mittal et al., 2013).

Several reports pertaining to the plant-mediated synthesis of AgNPs have been published. These reports have demonstrated the efficiency of plant metabolites in the synthesis of bioactive AgNPs. Although plant-mediated synthesis of AgNPs has been demonstrated, it is important to note that each plant species has its unique composition of bioactive constituents. These plant secondary metabolites include flavonoids, phenolic compounds, glycosides, and sterols, which have been reported to be responsible for the reduction of silver ions to the elemental silver (Dauthal and Mukhopadhyay, 2016). Since plant species vary in the composition and quantity of secondary metabolites, a broad range of plant species should be examined for their ability and efficiency to biosynthesize metal nanoparticles. This exploration can be used to overcome biomedical constraints and other production-related problems. Synthesis of AgNPs using phyto-molecules may help to overcome the problems associated with toxic chemical reagents. The plant *Semecarpus anacardium* (family Anacardiaceae) is a well-known medicinal plant in India. In the Ayurvedic system of medical treatment, different plant parts are commonly used as a medicine for alimentary tract impairments and certain dermatological problems. *S. anacardium* has a positive effect on blood pressure, cancer, and heart disease, as well as respiration and neurological disorders (Mohanta et al., 2007). *Glochidion lanceolarium* (family Phyllanthaceae) is an important ethnomedical plant used for the treatment of gastrointestinal complaints (Chanda et al., 2007) and *Bridelia retusa* (family Phyllanthaceae) exhibits pharmacologically potent antiviral, hypoglycemic, and immunomodulatory activity (Ghawate et al., 2015). The latter plant has also been reported to be effective in wound healing (Deore et al., 2014). The anticancer properties of these plants, however, have not been fully explored. Since many cancer drugs exhibit debilitating side effects and have a questionable impact on cancer cells, the use of natural medicines derived from natural sources should be urgently explored.

The management of public health is a major issue in the modern, industrial world, due to the rising pervasiveness of microbial resistance. Although numerous new antibiotics have been discovered and developed, multidrug-resistant bacteria are still becoming more prevalent and are creating a serious public health risk (Mohanty et al., 2017).Thus, there is a critical need to develop a new, powerful therapeutic approach to treat and kill Gram-negative, as well as Gram-positive, human pathogens. The biosynthesis and application of metal nanoparticles is now receiving great interest as a viable option due to its moderate success in being used to facilitate drug delivery (Zhang et al., 2008), treat chronic disease (Hong et al., 2008), and for its ability to treat bacterial infections in wounded tissues (Rai et al., 2009).

Comprehensive studies of antimicrobial resistance have revealed that bacterial infections that resist antibiotics are not due to free bacteria but rather to bacteria existing within a biofilm (Liu et al., 2019). Biofilm-forming bacteria are resistant to conventional antimicrobials due to: (1) the inability of the antimicrobial to penetrate the biofilm, (2) evolution complex drug resistance properties, and (3) biofilm mediated inactivation or modification of antimicrobial enzymes (Elbourne et al., 2019). Fortunately, nanoparticle-based antimicrobials have been developed and marketed to eradicate both planktonic and biofilm-forming antibiotic-resistant bacteria. Continuous research is being conducted to develop eco-friendly nanotechnologies utilizing natural phytochemicals to produce metal nanoparticle-based antimicrobials for the control of biofilm-forming pathogens.

In this regard, the objective of the current study was to explore the use of ethno-medicinally important plants, *S. anacardium*, *G. lanceolarium*, and *B. retusa* (Panda et al., 2016), for the synthesis of AgNPs utilizing secondary metabolites present in leaf extracts. The formation of AgNPs were monitored by microscopy and spectroscopy to evaluate the synthesis process, and the phytoconstituents most likely responsible for the synthesis of the AgNPs compounds were characterized using different qualitative tests. The phyto-mediated synthesized AgNPs were also screened for antibacterial and anti-biofilm activity against the human pathogens, *Pseudomonas aeruginosa*, *Escherichia coli* (Gram –ve), and *Staphylococcus aureus* (Gram +ve) in order to assess their potential use in antimicrobial therapy.

## Materials and Methods

### Chemicals and Reagents

The chemicals used during the experiments, such as Mueller Hinton medium and silver nitrate (AgNO_3_), were purchased from Hi-media (India) and Sigma-Aldrich (India), respectively.

### Microbial Strains and Plant Specimens

The bacterial strains of *P. aeruginosa* (MTCC 741), *E. coli* (MTCC 739), and *S. aureus* (MTCC 96) were used in the present study. The species were obtained from microbial type culture collection, IMTECH, Chandigarh, India and stored in the culture collection located in the Department of Botany, North Orissa University, India. The plants used for the phyto-assisted synthesis of AgNPs were collected from the Simlipal Biosphere Reserve, India. Before beginning the experiments, proper identification and deposition of plants were conducted in the Department of Botany, North Orissa University with allocation of voucher specimen numbers *S. anacardium* (NOU KL 024/2014), *G. lanceolarium* (NOU KL 085/2014), and *B. retusa* (NOU KL 080/2014).

### Qualitative Phytochemical Analysis

The qualitative phytochemical analysis of *S. anacardium*, *G. lanceolarium*, and *B. retusa* extract was performed by following the standard method as reported by Arunachalam et al. (2012), with slight modifications. The obtained results were qualitatively expressed as positive (+) or negative (−) (Guruvaiah et al., 2012). The chemicals and reagents used in the study were purchased from Sigma-Aldrich (India).

### Quantitative Phytochemical Analysis and *in vitro* Antioxidant Properties

#### Total Phenolic Content Determination

The total phenolic quantity, in the leaf extracts of *S. anacardium*, *G. lanceolarium*, and *B. retusa* were measured using the standard Folin–Ciocalteu method with required modifications (McDonald et al., 2001). All the experiments were performed in triplicate and the total phenolic content was expressed as gallic acid equivalent (GAE) in mg/g sample.

#### Total Flavonoids Content Determination

The total amount of flavonoids in the leaf extracts of *S. anacardium*, *G. lanceolarium*, and *B. retusa* was estimated by the standard aluminum chloride method with required modifications (Chang et al., 2002). All the estimations were carried out in triplicate and total flavonoid content was expressed as GAE in mg/g sample.

#### 1,1-Diphenyl-2-Picryl-Hydrazil Radical Scavenging Activity

Potential antioxidant activity of leaf extracts of *S. anacardium, G. lanceolarium, and B. retusa* was determined using 1,1-diphenyl-2-picryl-hydrazil (DPPH) assay with required modifications (McDonald et al., 2001). Various concentrations, such as 10, 20, 30, 40, and 50 mg/ml extracts, were taken for the study of DPPH scavenging activities. The results were expressed as percentage (%) radical scavenging activity. The minimum inhibitory concentration (MIC) was calculated and results were presented IC_50_ value. The equivalent concentrations of ascorbic acid were taken as a positive control.

### Sample Preparation and Synthesis of Silver Nanoparticles

Fresh leaves were collected from healthy plants of. *S. anacardium*, *G. lanceolarium*, and *B. retusa* growing in a hilly area of the Simlipal Biosphere Reserve, India. The plant leaves were thoroughly washed and then dried in a hot air oven. The dried leaves were then pulverized and passed through a 20-mesh sieve. Five gram of leaf powder were added to 50 ml of sterilized deionized water and sonicated for 15–20 min. The resulting mixture was filtered through Whatman’s filter paper and maintained at 4°C until further use. Extracts were prepared from each of the species separately. The filtered leaf extracts were used to synthesize the AgNPs by adding 10 ml of extract to 90 ml of 1 mM AgNO_3_ in an aqueous solution and incubating the resulting solution overnight at 60°C. A similar protocol was used for each of the plant extracts. The synthesis of AgNPs was monitored by UV–Vis spectrophotometry (Perkin Elmer-λ35) in the range of 350–600 nm.

### Characterization of Silver Nanoparticles

The synthesized AgNPs were characterized using methods that have been previously described (Mohanta et al., 2016a). The size range and surface charge (Zeta) of dispersed NPs were determined using a Zeta sizer (Nano ZS90, Malvern Instruments Ltd, Malvern, United Kingdom). Attenuated total reflectance-Fourier transform infrared spectroscopy (ATR-FTIR, Brucker) analysis of spectra within the range of 500–4,000 cm^–1^ was conducted to determine the role of the phytoconstituents in NPs synthesis. Surface morphology was confirmed by observation of the synthesized AgNPs with a Field emission scanning electron microscope (FE-SEM; Jeol 6480LV JSM microscope, United States) operating at an acceleration voltage of 15 KV.

### Antibacterial Activity and Minimum Inhibitory Concentration Evaluation of AgNPs

The different species of pathogenic bacteria were cultured in Mueller Hinton Broth (MHB) to determine the MIC of the synthesized AgNPs. Cell suspensions of each organism were adjusted to attain the required cell numbers per ml by measuring the turbidity of cell suspensions in a spectrophotometer (Perkin Elmer, Lambda35, Germany). The antimicrobial assay was conducted in 96-well microtiter plate using a two-fold serial dilution of AgNPs to determine 50% inhibition of microbial growth. Standard broth (MHB) was used according to the Clinical and Laboratory Standards Institute (CLSI) guidelines (Clinical, and Laboratory Standards Institute, 2005). In addition to the synthesized AgNPs, the extracts from the respective plants were also assayed for antimicrobial activity. A standardized concentration of each test organism was obtained by adjusting the turbidity of each suspension culture to an OD = 0.003 at 600 nm (∼100-fold dilution of parent culture). For the assay, 190 μl of the test-adjusted cell suspension of an organism and 10 μl of different concentrations (μg/ml) of the AgNPs were added to wells of a microtiter plate. The same protocol was used to assay the plant extracts and the antibiotic Gentamycin, the latter of which was used as a positive control. To correct for the absorbance of the AgNPs or plant extracts, control wells were used containing 190 μl MH broth (devoid of organisms) and 10 μl of AgNPs or plant extract. The plates were wrapped with parafilm and incubated after a thorough mixing of the components. The MICs of AgNPs were expressed as an IC_50_ value. All experiments were carried out in triplicate and the mean ± standard deviation was calculated.

### Anti-biofilm Activity and Minimum Inhibitory Concentration Determination for the AgNPs

A 96-well microtiter plate (flat bottom, polystyrene) was used to determine the anti-biofilm activity of the AgNPs as described by Gurunathan et al. (2014) and Barapatre et al. (2016). Individual wells of the plates were filled with 180 μl of Muller Hinton Broth and 10 μl of the test pathogens (OD = 1.0, 600 nm) were added. Subsequently, 10 μl of AgNPs were added and the preparation was thoroughly mixed. A two-fold dilution series of the AgNPs was used to determine the MIC of AgNPs against biofilm formation. The same was used to test the anti-biofilm activity of the plant extracts and to determine their MIC. After completing the preparation of the test plates, they were incubated in a static condition for 24 h at 37°C. Mixtures without bacteria were used to adjust the OD for absorbance by the extract components. After incubation, the contents of the wells of the microtiter plates were discarded and gently washed with phosphate buffered saline (PBS, pH 7.2) to remove free-floating non-adherent bacterial cells from the walls and bottom of the wells. The wells of the microtiter plates were then air dried for 45 min. After drying, adherent “sessile” bacteria in the wells were fixed with 2% w/v sodium acetate and the wells were then flooded with crystal violet stain (0.1%, w/v) and incubated in the dark for 30 min. Afterward, the wells were thoroughly washed with sterile deionized water until all excess dye was removed. The plates were then air dried again. After complete drying, 200 μl of ethanol (95%, v/v) was added to each well and absorbance at 620 nm was measured (Multi-scan plate reader, Thermo Fisher scientific). The percentage of inhibition of biofilm formation was calculated using following equation:

(1) %biofilminhibition=[1-(ODo620fcellstreatedwithAgNPsorplantextracts/ODo620fthenon-treatedcontrol)×100].

All assays were conducted in triplicate and mean ± standard deviation was calculated. The MIC for anti-biofilm potential was expressed as an IC_50_.

### Synergistic Antibacterial and Antibiofilm Activity

The synergistic antibacterial and antibiofilm activity was studied by taking different combinations of pure leaf extract and as-synthesized AgNPs from *S. anacardium*, *G. lanceolarium, and B. retusa* following the microbroth dilution method and microtiter plate anti-biofilm assay, respectively.

## Results and Discussion

### Qualitative and Quantitative Phytochemicals Assessment and Their Antioxidant Activities

The qualitative and quantitative phytochemical examinations of the aqueous leaf extracts are summarized in Tables 1, 2. The qualitative phytochemical analysis revealed the existence of alkaloids, flavonoids, tannins, phenolic, proteins, and saponins in all three plants (*S. anacardium, G. lanceolarium*, and *B. retusa*) but resin, steroids, and sterols were not detected. Glycoside was present only in *S. anacardium* extract, whereas sugar was found in *B. retusa*. The phytochemical study of the leaf extract of these three plants showed that flavonoids, tannins, phenolic compounds, proteins, and saponins were present in the extract, which may be the principal chemical constituents responsible for the synthesis of AgNPs. Shankar et al. (2003) reported the possible role of terpenoids from *Geranium* leaf in the synthesis of nano-sized Ag particles. Polyols such as terpenoids, flavones, and polysaccharides in the *C. camphora* leaf were reported to be the main cause of the bio-reduction of silver and chloroaurate ions (Huang et al., 2007). The quantitative phytochemical results supported the possibility of a greater potential for antioxidant activity (Table 2). The plant extract possesses huge potential for providing natural chemicals for the reduction of complex metals to derive respective nanoparticles for diverse important applications.

**TABLE 1:** Qualitative phytochemical screening of aqueous extract.

Name of the phytoconstituents	Observation		
	
	*S. anacardium*	*G. lanceolarium*	*B. retusa*
Alkaloids	+	+	+
Tannins and phenolic compounds	+	+	+
Glycoside	+	−	−
Flavonoids	+	+	+
Steroids and sterols	−	−	−
Triterpenoids	+	+	+
Sugars	−	−	+
Resins	−	−	−
Proteins	+	+	+
Saponins	+	+	+

*+, present; −, absent.*

**TABLE 2:** Quantitative phytochemical constituents of aqueous extract.

Phytochemical constituent	mg/100 g dry weight (Mean ± SD)		
	
	*S. anacardium*	*G. lanceolarium*	*B. retusa*
Total phenol content	545.57 ± 25.00	330.57 ± 26.00	410.25 ± 22.15
Total flavonoid content	840.76 ± 24.10	510.76 ± 25.10	714.76 ± 25.10

**FIGURE 1:**
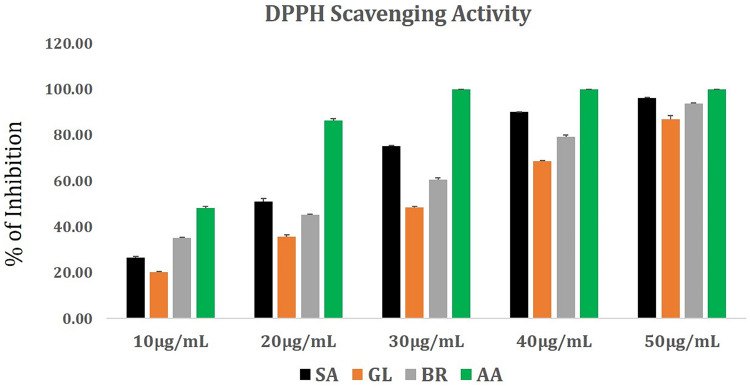
DPPH radical scavenging activity of plant extracts from *G. lanceolarium* (GL), *S. anacardium* (SA), *B. retusa* (BR). Ascorbic acid (AA) is taken as standard.

The antioxidant potential in terms of DPPH radical scavenging activity of *S. anacardium*, *G. lanceolarium*, and *B.retusa* positively responded toward the possible involvement of the antioxidant molecules from the leaf extract during the synthesis of AgNPs. It is well known that plants have a large repository of phenolics and flavonoids molecules which have super antioxidant capacity and are considered to be strong free radical scavengers. The DPPH radical scavenging result (Figure 1) revealed the antioxidant potentials of aqueous leaf extracts of three ethnomedicinally important plants. The percentage (%) DPPH scavenging activity (IC50) was found to be 16.98 ± 0.12 μg/ml, 30.71 ± 0.22 μg/ml, and 22.66 ± 0.25 μg/ml for *S. anacardium*, *G. lanceolarium*, and *B. retusa*, respectively. The presence of a significant concentration of total phenolics and flavonoids in *S. anacardium*, *G. lanceolarium*, and *B.retusa* leaves indicated a notable antioxidant activity. Previous research revealed that the high molecular weight and the adjacency aromatic rings and hydroxyl groups are more focused in the free radical scavenging activity of bioactive phyto-compounds (Hagerman et al., 1998; Luximon-Ramma et al., 2002; Pratap Chandran et al., 2013). The antioxidant activities were highly correlated with the total phenolic and flavonoids levels. It is also essential to evaluate the antioxidant potential as some of the plant molecules still remain with AgNPs post-purification as a capping and stabilizing agent, which should not be harmful to normal cells during the cellular application of the nanoparticles. Thus, the antioxidant potential of *S. anacardium*, *G. lanceolarium*, and *B. retusa* established that the green synthesis process of AgNPs is highly safe for biomedical applications.

### Synthesis and Characterization of AgNPs

The growing increase in microbial resistance to both currently available and newly developed antibiotics is a worldwide problem. Therefore, it is imperative that researchers identify and develop new methods to deal with antibiotic-resistant microbial pathogens, as well as the ever-growing appearance of new, virulent pathogens (Veerasamy et al., 2011). Currently, there is significant interest in the development of nanoparticles-based antibacterial agents to address the critical health problems brought about by the rapid evolution of drug-resistant microorganisms.

Silver has been recognized as an anti-infection agent since ancient times. Extensive research has been conducted on silver ions and silver conjugated salts as potential antimicrobial and anti-biofilm agents. Although silver has been used as a therapeutic agent, silver ions exhibit a level of toxicity and have low efficacy due to their inactivation caused by easily complexing with other molecules and their precipitation with other interfering salts (Mohanty et al., 2017). New strategies to address these problems are being explored, such as the use of leaf extracts of ethnomedicinal plants to synthesize AgNPs in a cost effective, bio-compatible, and eco-friendly manner.

The primary objective of the current study was to synthesize small-sized AgNPs using leaf extracts of the medicinal plants, *S. anacardium*, *G. lanceolarium*, and *B. retusa*, as a reducing, stabilizing, and capping agent. Silver nitrate (1 mM) was added to leaf extracts obtained from these plants and incubated overnight (60°C, pH 7.5) to regulate the size of particles. Successful synthesis of AgNPs was confirmed by visual observation of a color change of the solution, in which the pale-yellow color of the mixture of leaf extract and AgNO_3_ turned to a deep brown color. No color change was observed in control mixtures consisting of just leaf extracts and sterile, deionized water. The appearance of a deep brown color indicates the formation of AgNPs (Gurunathan et al., 2014).

**FIGURE 2:**
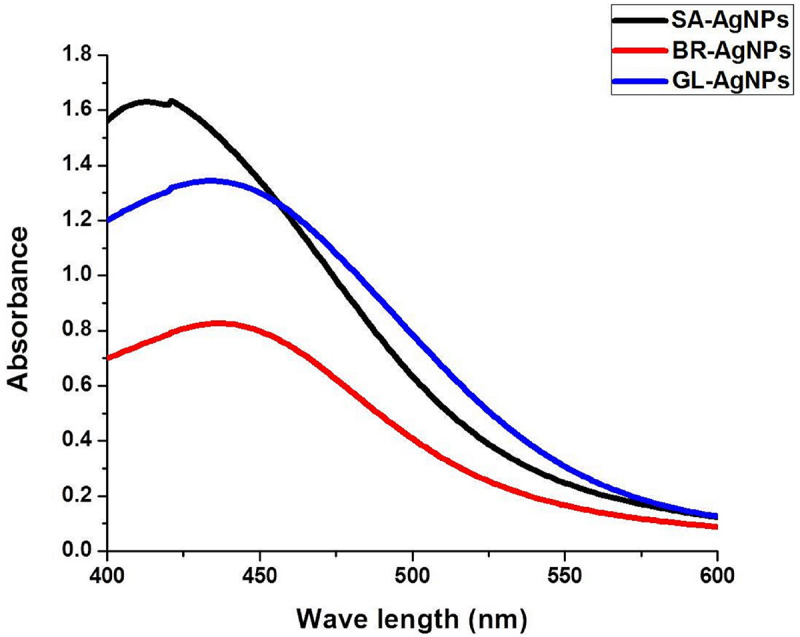
UV–Vis spectrophotometric analysis of silver nanoparticles.

Prior to determining the antimicrobial activity of the AgNPs, a detailed characterization of the synthesized AgNPs was conducted using methods described in our previous publications (Mohanta and Behera, 2014; Mohanta et al., 2017a, b). UV–visible spectroscopy is an established method for the study of metal nanoparticles. We used UV–Vis spectrophotometric analysis to monitor the continuous synthesis of AgNPs. A strong and broad peak within the 420–430 nm range was frequently observed, which is the peak range characteristic for AgNPs (Figure 2). This unambiguous and characteristic peak is created due to the SPR of the particles, a characteristic that has been widely established and recognized for different metal nanoparticles in the 2–100 nm size range (Nayak et al., 2015). Notably, the nanoparticles were synthesized by the addition of 10 ml of leaf extract to 90ml of a solution of AgNO_3_.

The color of the reaction mixture turned from a pale yellow to a deep brown after an appropriate length of incubation, indicating the formation of AgNPs. It is believed that the change in color of the reaction mixture is due to the presence of specific phytocompounds in the leaf extract, suggesting that the reducing power of the phytoconstituents is responsible for the synthesis of the AgNPs. If so, this represents a novel, eco-friendly approach to the synthesis of metal nanoparticles. The absorption peak characteristic of AgNPs was clearly detected due to the combined vibration of the electrons of AgNPs being in resonance with the specific wavelengths of light. The distinct and single SPR band at 420–430 nm confirms the reduction of Ag^+^ to Ag^0^. Similar observations have been made in studies on the synthesis of AgNPs (Krishnaraj et al., 2010; Gopinath et al., 2012; Iravani and Zolfaghari, 2013).

### Particle Size Distribution and Surface Charge Analysis of AgNPs

**FIGURE 3:**
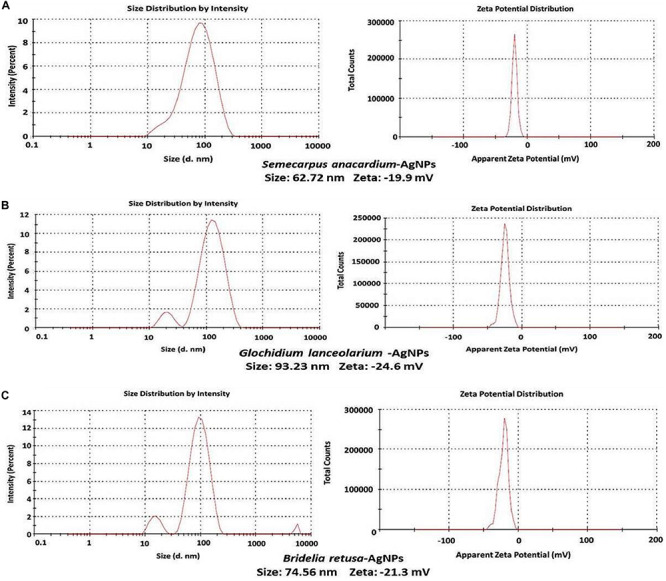
DLS analysis of AgNPs synthesized using plant extracts of **(A)** *Semecarpus anacardium*, **(B)** *Glochidion lanceolarium*, or **(C)** *Bridelia retusa*.

It was important to determine the particle size and charge of the synthesized AgNPs in aqueous solution prior to assessing their antimicrobial and anti-biofilm activity. Particle size, surface charge, morphology, and particle composition are the major factors that determine the *in vitro* toxicity of AgNPs (Bhanumathi et al., 2017). A dynamic light scattering spectroscopy (DLS) analysis was conducted to measure particle size and charge in an aqueous solution. This technique allows for the rapid determination of particle size distribution and surface charge of nanoparticles in solution (Lim et al., 2013). Results of the DLS analysis revealed that the average particle sizes of the AgNPs synthesized from the three plant extracts were 62.72, 93.23, and 74.56 nm for *S. anacardium* (SA-AgNPs), *G. lanceolarium* (GL-AgNPs), and *B. retusa* (BR-AgNPs), respectively (Figures 3A–C). Particle sizes <100 nm have greater potential in biomedical applications, as the type of interaction that occurs between nanoparticles and cells is highly dependent on the size of the nanoparticle. Surface charge is another crucial aspect of nanoparticles that affects their ability to associate with or complex with macromolecules present on the surface or inside cells. Thus, the charge or Zeta potential of the AgNPs synthesized using the three different plant extracts was assessed to determine their potential to interact with biological macromolecules. Results indicated a charge of -19.9, -24.6, and -21.3 mV for the AgNPs synthesized using extracts of *S. anacardium*, *G. lanceolarium*, and *B. retusa*, respectively (Figures 2A–C). Several studies have been previously reported on the particle size and charge of AgNPs that support our results (Guruvaiah et al., 2012; Gurunathan et al., 2014; Chung et al., 2016).

### Scanning Electronic Microscopic Analysis

**FIGURE 4:**
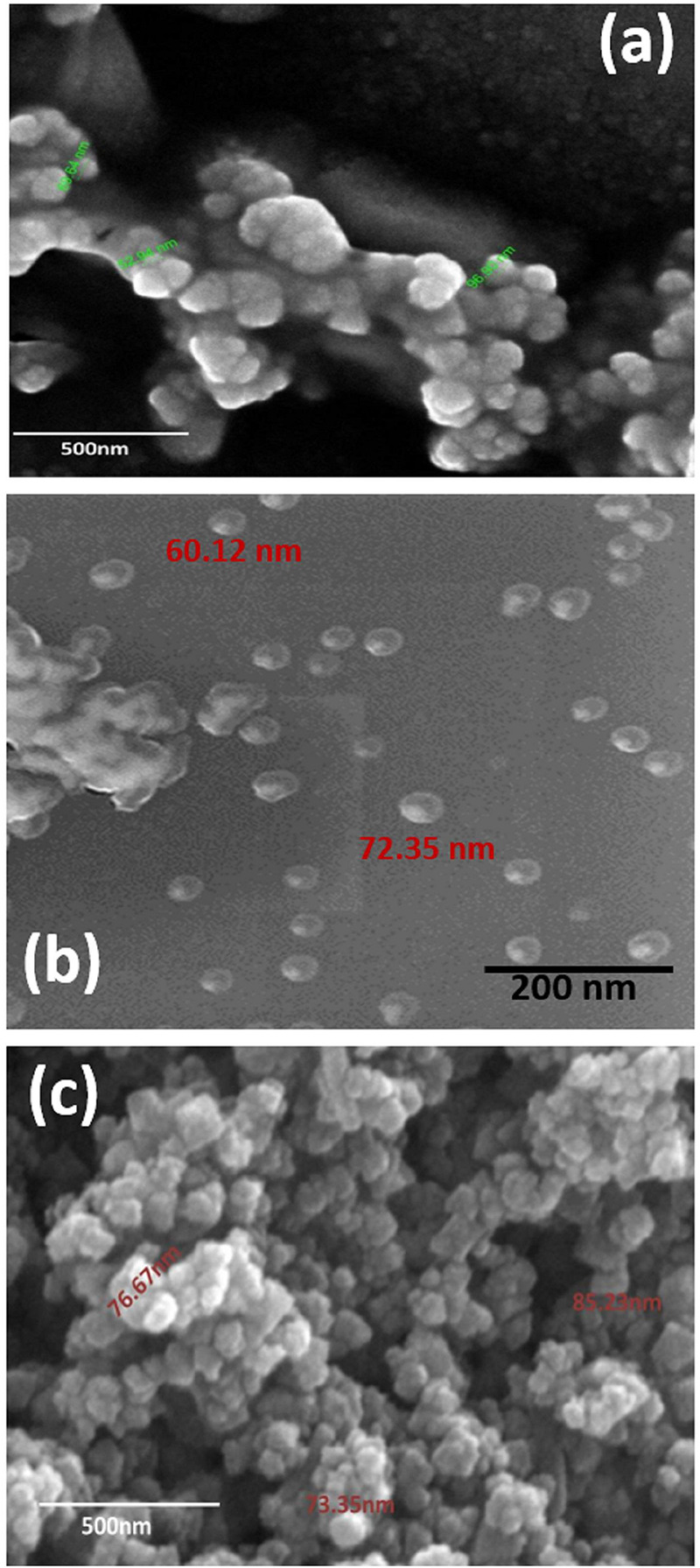
SEM micrograph of **(a)** GL-AgNPs, **(b)** SA-AgNPs, and **(c)** BR-AgNPs. GL indicates *Glochidion lanceolarium*, SA indicates *Semecarpus anacardium*, and BR indicates *Bridelia retusa*.

Scanning Electronic Microscope (SEM) is an invaluable tool for obtaining structural information about nanoparticles and was therefore used to characterize the size and morphology of the AgNPs synthesized in the present study. Results obtained from the analysis of SEM micrographs of the synthesized AgNPs indicated that the synthesized nanoparticles were distinct, uniform in shape, spherical, and well-separated. The average size of the particles ranged between 52 and 96 nm (Figures 4a–c).

### ATR-FTIR Analysis

**FIGURE 5:**
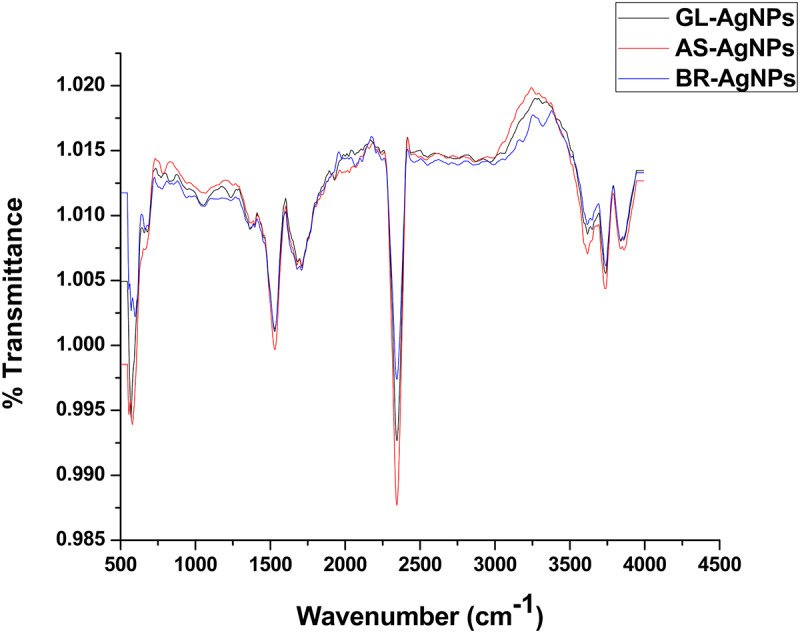
ATR-FTIR analysis of AgNPs.

The spectra obtained with Attenuated total reflectance Fourier transform infrared spectroscopy (ATR-FTIR) was used to classify the functional groups of the phytoconstituents present in the leaf extracts, which were potentially involved in the synthesis and stabilization of the synthesized silver nano-particles. The interaction of AgNPs with the phytoconstituents present in the leaf extracts of all three plant species exhibited intense peaks at 3735.93, 2247.27, 1537.60, 1717.06, and 570.99 cm^–1^ (Figure 5). A strong absorption peak at 3735.93 cm^–1^ strongly suggests the binding of silver ions with the hydroxyl (–OH) group stretching from water and the broad spectrum at 2247.27 cm^–1^ indicates a strong stretching of -C≡N (nitrile) group. The other three bands (∼1717.06, 1537.60, and ∼570.99 cm^–1^) were due to stretching vibrations of C=O (ketone), C=O (amide), and C–I functional groups. C=O (ketone) and C=O (amide) are generally present in proteins involved in the reduction of metal ions. These data suggest that hydroxyl and carbonyl groups may be responsible for the synthesis and stabilization of AgNPs. The IR data revealed that the compounds present in the leaf extract were present as a layer over the phyto-synthesized AgNPs and act as a stabilizing agent. Structural mechanism reveals that via Free amine groups or cysteine residues in the saponins, phenolics and quinones present in the leaf extracts have the ability to bind to the AgNPs and stabilize them through the surface-binding of a variety of plant compounds (Pant et al., 2012; Saranyaadevi et al., 2014).

### Antibacterial Activity of AgNPs

**FIGURE 6:**
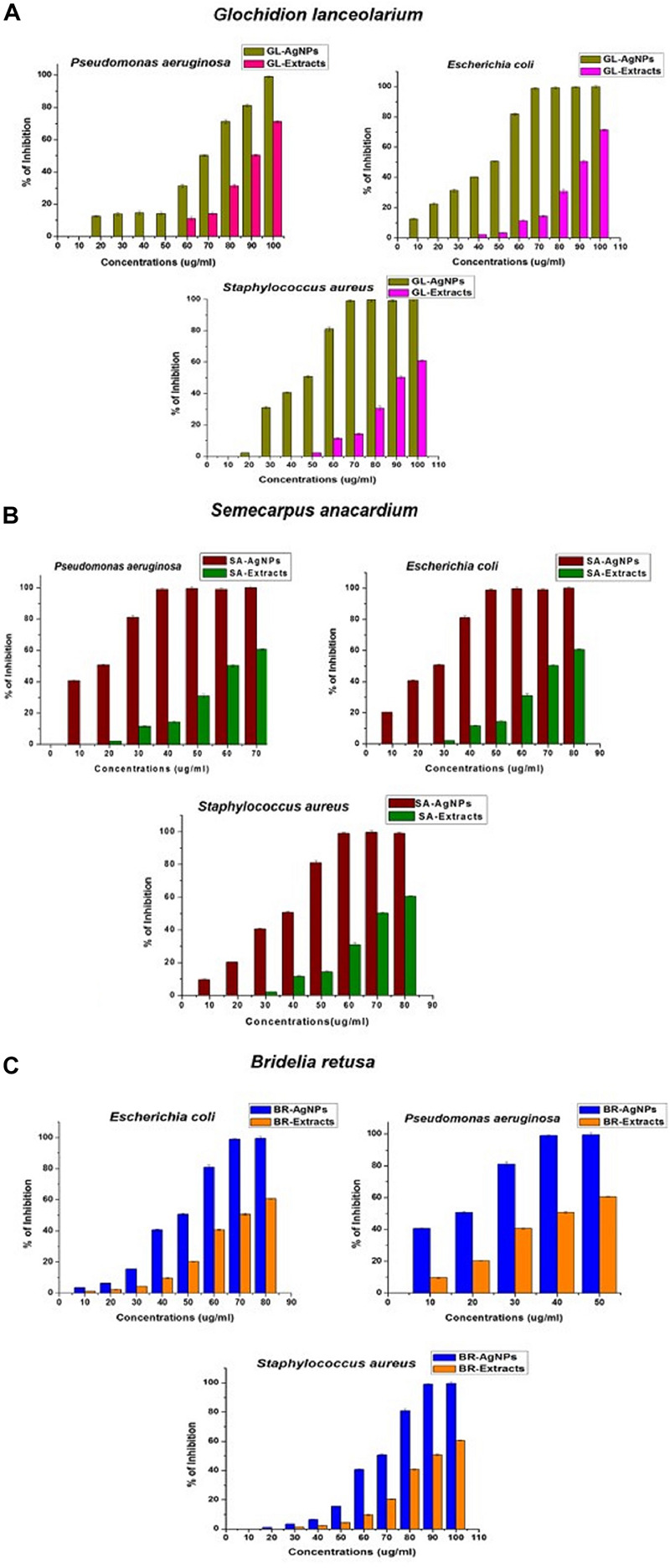
Antibacterial activity of AgNPs synthesized using plant extracts of **(A)** *Glochidion lanceolarium*, **(B)** *Semecarpus anacardium*, or **(C)** *Bridelia retusa.*

The bactericidal activity of AgNPs synthesized using the three different plant extracts was determined against Gram-positive and Gram-negative bacteria and revealed a dose-dependent relationship. Figure 6 illustrates the toxic activity of the plant synthesized AgNPs <100 nm at different concentrations (10–100 μg/ml) against the Gram-positive bacterium, *S. aureus*, and the Gram-negative bacteria *P. aeruginosa* and *E. coli*. Results indicate that the plant synthesized AgNPs can inhibit bacterial growth *in vitro*, relative to the growth of non-treated bacteria or the negative control. These data further indicate that the inhibition of bacterial growth increases with increasing concentrations of the AgNPs. Each species of bacteria was inhibited in their growth at their respective MIC value. The MIC values of the AgNPs synthesized from the three different plant extracts were calculated for each bacterial species. AgNPs exhibited excellent MIC (IC_50_) values against the different species irrespective of the source plant that was used in their synthesis. The MICs (IC_50_) of AgNPs synthesized using the leaf extract of *G. lanceolarium* was 43.94 ± 0.2μg/ml, 68.6 ± 0.5μg/ml, and 44.02 ± 0.3μg/ml against *S. aureus*, *P. aeruginosa*, and *E. coli*, respectively. Concentrations of 70 μg/ml (*S. aureus*), 100 μg/ml (*P. aeruginosa*), and 80 μg/ml (*E. coli*) resulted in >99% inhibition (Figure 6A). Similar results were observed with AgNPs synthesized with *S. anacardium* plant extract. The MICs (IC_50_) of these AgNPs were 33.77 ± 0.2 μg/ml,12.9 ± 0.2 μg/ml, and 23.49 ± 0.2 μg/ml for *S. aureus*, *P. aeruginosa*, and *E. coli*, respectively. *S. anacardium* derived AgNPs exhibited > 99% inhibition at concentrations of 60 μg/ml (*S. aureus*), 40 μg/ml (*P. aeruginosa*), and 50 μg/ml (*E. coli*) (Figure 6B). AgNPs synthesized using *B. retusa* plant extract also exhibited strong antibacterial activity against the test pathogens exhibiting MICs of 64.13 ± 0.3 μg/ml (*S. aureus*), 12.90 ± 0.2 μg/ml (*P. aeruginosa*), and 43.94 ± 0.2 μg/ml (*E. coli*). AgNPs synthesized with *B. retusa* plant extract exhibited >99% inhibition at 90, 50, and 70 μg/ml, against *S. aureus*, *P. aeruginosa*, and *E. coli*, respectively (Figure 6C). A comparison of the obtained inhibitory activity indicated that AgNPs synthesized using *G. lanceolarium* plant extract were more active against the Gram-positive bacterium (*S. aureus*) while the AgNPs synthesized using *S. anacardium* or *B. retusa* plant extracts exhibited stronger antibacterial activity against the Gram-negative bacteria, *P. aeruginosa* and *E. coli*. Although, AgNPs derived from plant extracts of all three plant species inhibited both Gram positive and Gram-negative bacteria. AgNPs derived from *S. anacardium* plant extract had the strongest antibacterial activity and was active against both types of bacteria. Therefore, we suggest that *S. anacardium* plant extracts have the potential to be commercially used to synthesize AgNPs < 100 nm. Apart from the antibacterial activity of the derived AgNPs, *S. anacardium* is also an important medicinal plant, containing alkaloids and polyphenols with high medicinal value, which further strengthens its value in the production of bioactive nanoparticles. The individual plant extracts were also separately analyzed for bactericidal activity to compare their activity with the activity of AgNPs synthesized from their respective plant extracts. Results indicated that all of the plant-extract-derived AgNPs have greater antibacterial activity then the use of their respective plant extracts alone, further confirming the values of using plant-derived AgNPs as antibiotic compounds against human bacterial pathogens.

The AgNPs synthesized using plant extracts exhibited significantly greater antibacterial activity than AgNPs synthesized from other sources, such as bacteria, fungi, algae, etc. Multiple reports support the use of AgNPs as antibacterial agents (Rai et al., 2009; Ahmad et al., 2015; Kotakadi et al., 2015; Abbasi et al., 2016; Nayak et al., 2016) and several mechanisms have been proposed regarding the antibacterial activity of AgNPs. Earlier studies by Sondi and Salopek-Sondi (2004) focused on the interaction of AgNPs with *E.coli* and confirmed that at the first stage of interaction, AgNPs attach to the bacterial cell wall. After stable adherence, AgNPs penetrate the bacterium and induce cell death by rupturing the cell membrane. AgNPs acting as oxidizing agents on the surface of proteins present on the plasma membrane and cellular homeostasis have also been suggested as the mechanism underlying AgNP antibacterial activity. Another suggestion is that AgNPs attach to the cell membrane surface and decrease its permeability and respiration. The current study, although supportive of all the proposed mechanisms, confirms that the bactericidal activity involves the uptake of AgNPs. Notably, plant-synthesized AgNPs have an added advantage over chemically synthesized AgNPs due to the ability of plant metabolites to function as capping and stabilizing agents, as well as exhibiting their own antibacterial activity, which collectively enhances the antibacterial activity of AgNPs.

### Anti-biofilm Activity of AgNPs

**FIGURE 7:**
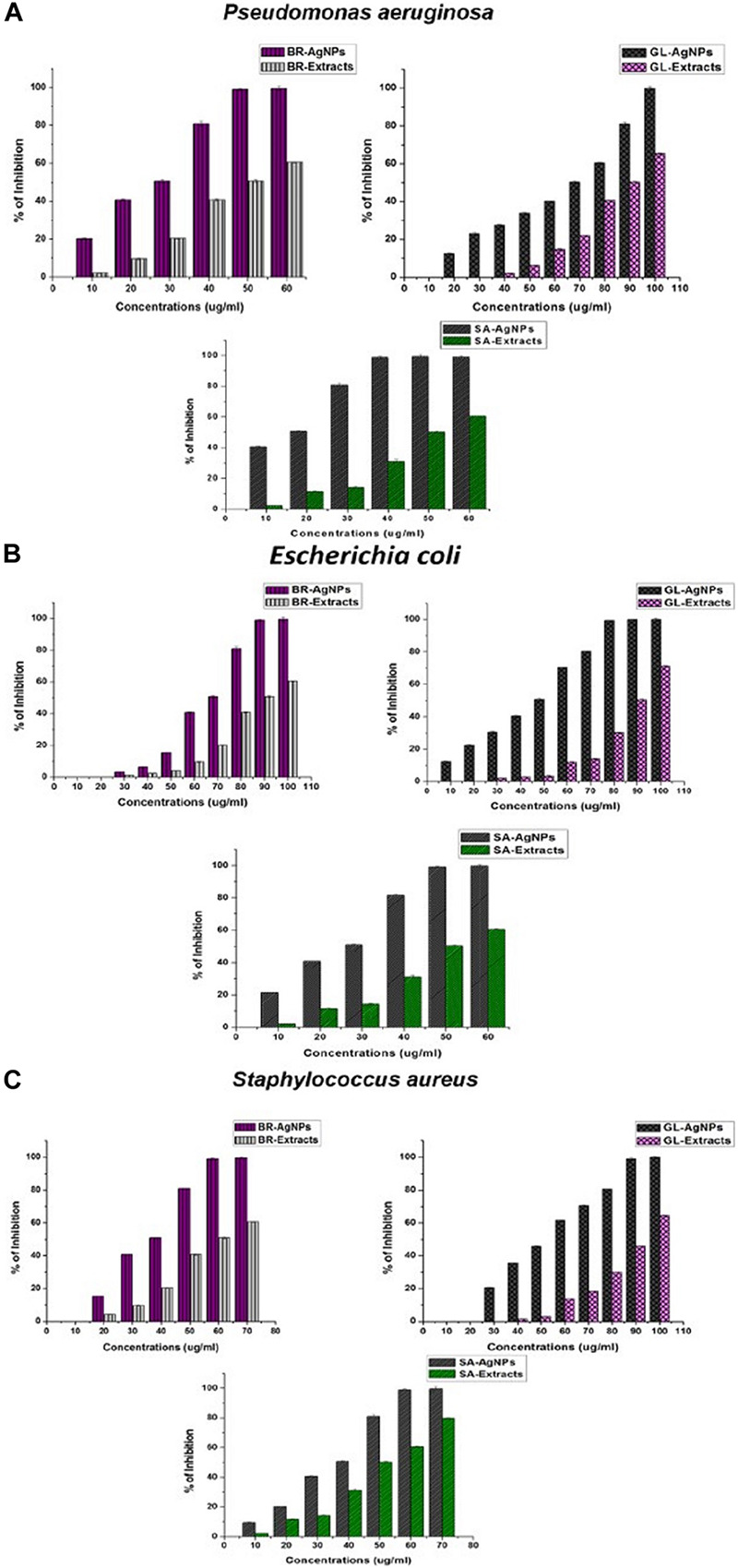
Anti-biofilm activity of AgNPs against **(A)** *Pseudomonas aeruginosa*, **(B)** *Escherichia coli*, and **(C)** *Staphylococcus aureus* synthesized by the plant extracts (*G. lanceolarium*, *S. anacardium*, and *B. retusa*).

Silver nanoparticles have also been assayed for anti-biofilm activity against biofilm-forming bacteria. In the present study, the *in vitro* anti-biofilm activity of AgNPs was evaluated in a dose-dependent manner against the biofilm- forming bacteria *P. aeruginosa*, *E. coli*, and *S. aureus.* The individual species of bacteria were grown in 96-well microtiter plates for 24 h and then treatments of 10–100 μg/ml of the individually synthesized AgNPs were added to each well. Results of the assay revealed that the biosynthesized AgNPs inhibited biofilm formation by the bacterial species, relative to the negative control used in the experiment (Figures 7A–C). The MICs of anti-biofilm activity was expressed in terms of IC_50_ and all AgNPs, irrespective of which extract was used in their synthesis, exhibited an excellent MIC value against bio-film formation.

Treatment of *P. aeruginosa* for 24 h with AgNPs (100 μg/ml) synthesized using *G. lanceolarium* plant extract, reduced biofilm formation by >99%. Similarly, AgNPs derived from *S. anacardium* and *B. retusa* at a concentration of 50and 60 μg/ml, respectively, reduced biofilm formation by >99%. The MIC (IC_50_) of AgNPs needed to inhibit biofilm formation were 68.94 ± 0.2 μg/ml, 12.9 ± 0.2 μg/ml, and 23.48 ± 0.2 μg/ml for AgNPs derived from *G. lanceolarium, S. anacardium*, and *B. retusa*, respectively. AgNPs derived from *S. anacardium* plant extract exhibited potent inhibition of biofilm formation at a very low IC_50_ value. AgNPs derived from *B. retusa* also exhibited high levels of inhibition of biofilm formation against *P. aeruginosa.* Notably, the AgNPs derived from all three plant sources exhibited anti-biofilm activity against *E. coli*. concentrations of 80, 50, and 100 μg/ml. AgNPs derived from *G. lanceolarium, S. anacardium*, and *B. retusa*, respectively, exhibited >99% inhibition against biofilm formation by *E. coli*, a Gram-negative bacterium. MIC (IC_50_) values of 45.5 ± 0.2 μg/ml, 23.42 ± 0.15 μg/ml, and 64.14 ± 0.3 μg/ml were calculated for the AgNPs derived from *G. lanceolarium, S. anacardium*, and *B. retusa*, respectively. In the case of the Gram-positive bacterium, *S. aureus*, >99% inhibition was evident at 90 μg/ml (*G. lanceolarium*), 60 μg/ml (*S. anacardium*), and 60 μg/ml (*B. retusa*). The AgNPs derived from both *S. anacardium* and *B. retusa* greatly inhibited biofilm formation by *S. aureus.* The AgNPs also exhibited potent antibiofilm activity against *S. aureus* with a calculated MIC (IC_50_) of 52.54 ± 0.1 μg/ml (*G. lanceolarium*), 33.77 ± 0.2 μg/ml (*S. anacardium*), and 32.67 ± 0.15 μg/ml (*B. retusa*). The AgNPs derived from *S. anacardium* and *B. retusa* exhibited the best anti-biofilm activity against *S. aureus*.

Limited research has been conducted on the anti-biofilm activity of AgNPs. The formation of bacterial biofilms is the result of the synthesis and secretion of exopolysaccharides (EPSs) by bacterial cells, a prerequisite for biofilm formation (Landini et al., 2010). The bacteria respond to environmental cues that induce the synthesis of EPS. As a result, if the formation of EPS can be inhibited or prevented, then biofilm formation will also be restricted. This premise was the basis of our assay on the anti-biofilm activity of AgNPs. Kalishwaralal et al. (2010) previously reported the anti-biofilm activity of biosynthesized AgNPs against *P. aeruginosa* and *S. epidermidis.* In that study, AgNPs of 100 nm in size exhibited inhibited biofilm formation by 95-98%. Gurunathan et al. (2014) also studied anti-biofilm capacity of AgNPs against four human pathogens: *P. aeruginosa*, *Shigella flexneri*, *S. aureus*, and *Streptococcus pneumonia.* They reported high levels of anti-biofilm activity by AgNPs of a particle size < 100nm. Ansari et al. (2013) also investigated the anti-biofilm activity of AgNPs and clearly demonstrated that the inhibition of EPS synthesis was directly proportional to anti-biofilm activity. Park et al. (2010) studied the antibiofilm activity of AgNPs against *P. aeruginosa* and proposed that biosorption may be the major factor responsible for the inactivation of biofilm formation. Goswami et al. (2015) also reported on biofilm eradication by AgNPs and found that the use of 15 mg/ml of AgNPs resulted in an 89% inhibition of biofilm formation in *S. aureus* and 75% in *E. coli.* Results of the present research confirm the efficacy of Ag-NPs against biofilms produced by the Gram-negative bacteria, *P. aeruginosa* and *E. coli*, and the Gram-positive bacterium, *S. aureus*, at reasonably low concentrations. The current results also revealed that the tested bacteria are highly sensitive to GL-AgNPs, BR-AgNPs, and SA-AgNPs, suggesting that the complex biofilm signaling mechanism could also be associated with cell survival. Recently, research has been conducted on conjugating the antibiotic, rifampicin, with chemically synthesized AgNPs for use in combatting biofilm formation by methicillin resistant *S. aureus* and *Klebsiella pneumoniae* (Farooq et al., 2019).

### Synergistic Potential of Antibacterial and Antibiofilm Activity

**FIGURE 8:**
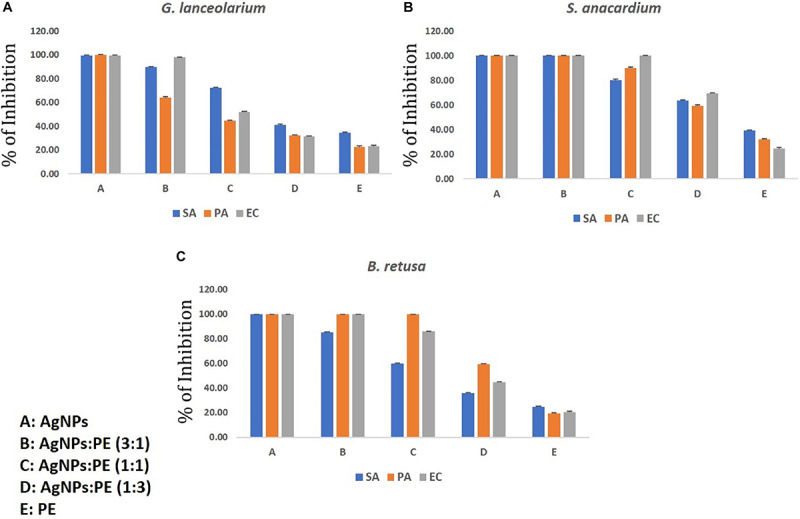
Synergistic antibacterial activity of AgNPs synthesized using plant extracts of **(A)** *Glochidion lanceolarium*, **(B)** *Semecarpus anacardium*, or **(C)** *Bridelia retusa.*

**FIGURE 9:**
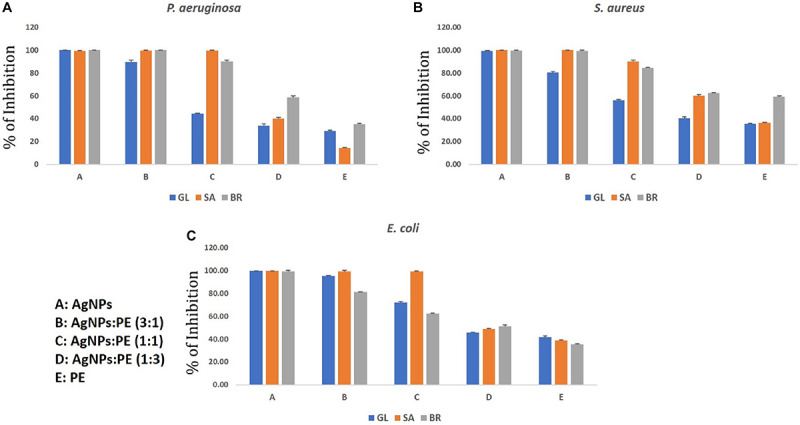
Synergistic anti-biofilm activity of AgNPs against **(A)** *Pseudomonas aeruginosa*, **(B)** *Staphylococcus aureus*, and **(C)** *Escherichia coli* synthesized by the plant extracts (*G. lanceolarium*, *S. anacardium*, and *B. retusa*).

The individual studies of antibacterial and antibiofilm activities on plant extract and synthesized AgNPs from the respective plant extracts have shown they have the reducing capacity to form respective bioactive AgNPs. The main aim of the synergistic activity study was to understand the possible combinatorial potentiality of the AgNPs and plant extract to control the biofilm formation by the pathogenic organisms. As discussed, the plant constituents having a reducing capacity if silver metallic salts are involved and attached to the AgNPs to stabilize the particles. During the synergistic study, the different ratios of synthesized AgNPs and plant extracts were mixed and applied for the antibacterial and antibiofilm activities and measured in terms of the percentage (%) of inhibition against the microorganisms taken for the study. The synergistic results are quite relevant to the results of the individual results, but overall synergistic potentiality was not that accountable what the individual activities were shown by AgNPs. Hence the current results of the synergistic activity are not recommended for practical applications. The results of both AgNPs and respective plant extracts synergistically are depicted in Figures 8, 9. The total concentration of the test samples were kept at 100 μg/ml and made the same with AgNPs and plant extracts with different ratios and tested against the test strains. Overall, the synthesized AgNPs alone have greater potential than the addition of respective plant extracts for controlling bacterial growth and biofilm formations. It can be assumed that when the concentration of the raw plant extract was increased, the antibacterial and antibiofilm activity was reduced due to the presence of some microbial growth promoting plant constituents in the extract. On the contrary, when the plant-synthesized AgNPs were applied, the results were highly significant for the purpose of the current study which revealed that the limited bioactive phytoconstituents with silver was highly active for the real world application in controlling the bacterial contaminations. In the antibacterial activity of GL-AgNPs (Figure 8A), it showed a greater potential effect against *S. aureus, P. aeruginosa*, and *E. coli*, while AgNPs showed reduced activities. When increasing concentrations of plant extract were added, the activity was lowered compared to the AgNPs. Therefore, the GL-AgNPs were shown to be highly promising for antibacterial activity rather than for synergistic applications. Likewise, in the case of SA-AgNPs (Figure 8B) and BR-AgNPs (Figure 8C), the individual AgNPs possessed greater potential compared to the synergetic activity. The antibacterial activity was reduced when the concentration of GL-AgNPs was low compared to GL-extracts against all *S. aureus, P. aeruginosa*, and *E. coli*. At a concentration of 100 μg/ml of GL-AgNPs, >99% inhibition was observed against all the pathogens, but when the concentration was reduced to a 1:3 ratio, the inhibition was 41, 32.10, and 31.4% against *S. aureus, P. aeruginosa*, and *E. coli*, respectively. Here it is clearly indicated that the GL-extract contains some nutritional factors for the microbes. In the case of SA-AgNPs, the 100% inhibition was found at a ratio of 3: 1 (AgNPs:SA-extracts) against all *S. aureus, P. aeruginosa*, and *E. coli*, but the percentage of the inhibition was gradually reduced when the concentration of SA-AgNPs was decreased. Likewise, BR-AgNPs possess the potential to act against test strains with 99.8% inhibition against *S. aureus* by SA-AgNPs solely. A more interesting result was found against *P. aeruginosa* where 100% inhibition was observed at the concentration of 1: 1 (BR-AgNPs: BR-extracts). At a 3:1 ratio of BR-AgNPs and BR-extracts, 100% inhibition was found against *E. coli*. From the results, the AgNPs are shown to have a better effect than mixing with respective plant extracts. For all the microorganisms used in the study, the phyto-synthesized AgNPs alone had better results compared with adding respective plant extracts for an antibacterial purpose. As the study was targeted to promote the natural plant product-based synthesis and applications of nanoparticles, the synergetic combination was focused by using plant extracts rather than any commercial antibiotics used against the organisms. Many synergetic applications of the AgNPs, along with current commercial antibiotics with different concentrations, show more antibacterial activities (Martinez-Gutierrez et al., 2013; Hussain et al., 2019; Rolim et al., 2019).

Similar results were observed in the case of the synergistic antibiofilm activity where as-synthesized AgNPs were more potentially active than the results found when the respective plant extracts was added in different concentrations. The reduced activity with the addition of plant extracts reveals the presence of microbial growth promoting factors in the natural plant extracts, as well as little antagonistic activity. Hence, the phyto-synthesized AgNPs are more promising than the synergistic application for controlling biofilm formations. Against all the three bacteria (*S. aureus, P. aeruginosa*, and *E. coli*), the respective AgNPs are more effective than the addition of respective plant extracts. The pure plant extracts were least active compared to the respective synthesized AgNPs. The lowest potential inhibition by the plant extracts reflects the presence of biofilm promoting entities. In the current study, *P. aeruginosa* was least inhibited by the SA-extracts (14%) which implies the extracts were supporting the biofilm formations. In other extracts, like GL-extracts and BR-extracts, the inhibition rate was 29.06 and 35.23%, respectively. In the case of *S. aureus* and *E. coli*, the inhibition was >95% in the ratio of 3(AgNPs: plant extracts) whereas <45% inhibition was observed in a 1:3 (AgNPs:plant extracts) ratio, which clearly showed least role of pure plant extracts in eradicating biofilm formation. The antibiofilm activity of AgNPs against *Acinetobacter baumannii*, *P. aeruginosa*, *S. aureus* (MRSA), *Streptococcus mutans*, and *Candida albicans* was studied extensively and reported the potentiality of AgNPs (Martinez-Gutierrez et al., 2013). Rolim et al. (2019) reported the antibiofilm activity of AgNPs against *P. aeruginosa* which supports our current results. However, the plant extract also has some potential of antimicrobial activity; these contain some microbial growth factors which help in growing, rather than inhibiting, the microbes. Hence, the AgNPs synthesized by the plant extracts were more promising than the sole plant extract and combination of plant extracts. The upcoming application of nanoparticles is highly promising as the uncontrolled spread of microbial contaminations is now a great threat worldwide.

## Conclusion

In the present study, plant extracts, derived from three different medicinal plant species, were used to synthesize AgNPs. The use of the plant extracts has an advantage over chemical or physical synthesis of AgNPs due to their ability to stabilize AgNPs, their own antibacterial properties, their high level of efficacy, and their low toxicity. Their use represents an eco-friendly approach to the synthesis of AgNPs. The extracts of three new plant resources (*S. anacardium*, *G. lanceolarium*, and *B. retusa*) were found to be have excellent potential for the commercial production of AgNPs. The plant-derived AgNPs exhibited strong antibacterial and anti-biofilm activity against different clinically important human pathogens. It is safer and more advantageous to biosynthesize metal nanoparticles using the natural reducing agents present in plants and microbes along with the conjugation of AgNPs to natural, antimicrobial molecules produced by microbes or plants. This line of research should be more greatly explored in the biomedical sciences. There is a high level of recently developed, drug-resistant microbes appearing in semi-tropical environments due to climate change, and this represents a significant health issue. Current antibiotics, and the antibiotic concept of fighting pathogens, is rapidly becoming insufficient for combatting new strains of existing pathogens and new disease-causing organisms. High throughput nanobiotechnology curated antimicrobials offer a new approach to treat microbial pathogens that are resistant to current treatment practices and for the treatment of biofilms. The present study demonstrates the potential of using plant-derived AgNPs to inhibit biofilm formation for therapeutic treatments that represent a new method of effectively treating a variety of infectious diseases caused by pathogenic microbes.

## Data Availability Statement

The datasets generated for this study are available on request to the corresponding author.

## Author Contributions

YM was involved in the collection of plant materials, synthesis of Ag-NPs, antimicrobial and antibiofilm assays, and the preparation of the manuscript. KB and SJ helped in the characterization of Ag-NPs and drafting the manuscript. AH, EA_A, and TM were involved in editing and revising the manuscript. All the authors read and approved the final manuscript.

## Conflict of Interest

The authors declare that the research was conducted in the absence of any commercial or financial relationships that could be construed as a potential conflict of interest.
