# Corrigendum: Anti-biofilm and Antibacterial Activities of Silver Nanoparticles Synthesized by the Reducing Activity of Phytoconstituents Present in the Indian Medicinal Plants

**DOI:** 10.3389/fmicb.2020.01784

**Published:** 2020-09-11

**Authors:** Yugal Kishore Mohanta, Kunal Biswas, Santosh Kumar Jena, Abeer Hashem, Elsayed Fathi Abd_Allah, Tapan Kumar Mohanta

**Affiliations:** ^1^Department of Botany, North Orissa University, Baripada, India; ^2^Department of Biotechnology, Maulana Abul Kalam Azad University of Technology, Haringhata, India; ^3^Department of Biotechnology, North Orissa University, Baripada, India; ^4^Botany and Microbiology Department, College of Science, King Saud University, Riyadh, Saudi Arabia; ^5^Mycology and Plant Disease Survey Department, Plant Pathology Research Institute, Agriculture Research Center, Giza, Egypt; ^6^Plant Production Department, College of Food & Agricultural Sciences, King Saud University, Riyadh, Saudi Arabia; ^7^Natural and Medical Sciences Research Center, University of Nizwa, Nizwa, Oman

**Keywords:** phyto-synthesis, silver nanoparticles, medicinal plants, anti-bacterial activity, anti-biofilm activity

In the original article, there was an error in one of the grants numbers in the Acknowledgments section. The correct number for the Researchers Supporting Project, King Saud University is (RSP-2020/134). Furthermore, the authors would like to remove the grant number NO (RGP-271). The revised Acknowledgments section appears below.

The authors are very thankful to their respective institutes for providing research facilities. The authors would like to extend their sincere appreciation to the researchers supporting project number (RSP-2020/134), King Saud University, Riyadh, Saudi Arabia.

The authors would like to state that this does not change the scientific conclusions of the article in any way. The original article has been updated.

